# Medications for preventing hypertensive disorders in high-risk pregnant women: a systematic review and network meta-analysis

**DOI:** 10.1186/s13643-022-01978-5

**Published:** 2022-07-01

**Authors:** Tippawan Liabsuetrakul, Yoshiko Yamamoto, Chanon Kongkamol, Erika Ota, Rintaro Mori, Hisashi Noma

**Affiliations:** 1 Department of Epidemiology, Faculty of Medicine, Prince of Songkla University, Hat Yai, Songkhla Thailand; 2 Department of Health Policy, National Center for Child Health and Development, Setagaya-ku, Japan; 3 Department of Community Medicine and Preventive Medicine, Faculty of Medicine, Prince of Songkla University, Hat Yai, Songkhla Thailand; 4 Global Health Nursing, Graduate School of Nursing Science, St. Luke’s International University, Chuo-ku, Japan; 5 Graduate School of Medicine, Kyoto University, Kyoto, Japan; 6 Department of Data Science, The Institute of Statistical Mathematics, Tokyo, Japan

**Keywords:** Medications, Hypertension prevention, Hypertensive disorders in pregnancy, High-risk pregnant women, Network meta-analysis

## Abstract

**Objectives:**

To determine the relative effectiveness of medications for preventing hypertensive disorders in high-risk pregnant women and to provide a ranking of medications using network meta-analysis.

**Methods:**

All randomized controlled trials comparing the most commonly used medications to prevent hypertensive disorders in high-risk pregnant women that are nulliparity and pregnant women having family history of preeclampsia, history of pregnancy-induced hypertension in previous pregnancy, obstetric risks, or underlying medical diseases. We received the search results from the Cochrane Pregnancy and Childbirth’s Specialised Register of Controlled Trials, searched on 31st July 2020. At least two review authors independently selected the included studies and extracted the data and the methodological quality. The comparative risk ratios (RR) and 95% confidence intervals (CI) were analyzed using pairwise and network meta-analyses, and treatment rankings were estimated by the surface under the cumulative ranking curve for preventing preeclampsia (PE), gestational hypertension (GHT), and superimposed preeclampsia (SPE). Safety of the medications is also important for decision-making along with effectiveness which will be reported in a separate review.

**Results:**

This network meta-analysis included 83 randomized studies, involving 93,864 women across global regions. Three medications, either alone or in combination, probably prevented PE in high-risk pregnant women when compared with a placebo or no treatment from network analysis: antiplatelet agents with calcium (*RR* 0.19, 95% *CI* 0.04 to 0.86; 1 study; low-quality evidence), calcium (*RR* 0.61, 95% *CI* 0.47 to 0.80; 13 studies; moderate-quality evidence), antiplatelet agents (*RR* 0.69, 95% *CI* 0.57 to 0.82; 31 studies; moderate-quality evidence), and antioxidants (*RR* 0.77, 95% *CI* 0.63 to 0.93; 25 studies; moderate-quality evidence). Calcium probably prevented PE (*RR* 0.63, 95% *CI* 0.46 to 0.86; 11 studies; moderate-quality evidence) and GHT (*RR* 0.89, 95% *CI* 0.84 to 0.95; 8 studies; high-quality evidence) in nulliparous/primigravida women. Few included studies for the outcome of superimposed preeclampsia were found.

**Conclusion:**

Antiplatelet agents, calcium, and their combinations were most effective medications for preventing hypertensive disorders in high-risk pregnant women when compared with a placebo or no treatment. Any high-risk characteristics for women are important in deciding the best medications. The qualities of evidence were mostly rated to be moderate.

**Systematic review registration:**

PROSPERO CRD42018096276

**Supplementary Information:**

The online version contains supplementary material available at 10.1186/s13643-022-01978-5.

## Background

Hypertensive disorders in pregnancy (HDP) are one of the five common complications during pregnancy, causing maternal and fetal deaths globally. The incidence of HDP ranges from 1 to 35% worldwide, with a wide variation across regions [1–3]. Due to the lack of a clear understanding of the underlying etiology of HDP, the antiplatelet agents, anticoagulants, antioxidants, nitric oxide, and calcium, which have been widely studied for their possible use in reducing or preventing HDP, were systematically reviewed [4–9]. To date, there have been two network meta-analyses. One was a conference abstract, which reported that calcium supplements reduced the risk of preeclampsia (PE) compared to aspirin, fish oil, and vitamin C or E [10]. Additionally, another network meta-analysis found that either vitamin D or calcium supplements may be effective [11].

A recent study demonstrated the superiority of Doppler and serum markers over conventional risk factor-based screening [12], and a new screening algorithm has recently demonstrated the effectiveness of aspirin prophylaxis in high-risk women [13, 14]. However, aspirin has been shown to be effective for high-risk women not only based on new screening algorithms but also on more traditional ways of defining high-risk, as shown in a Cochrane review [4]. In addition, these screening methods require experienced technicians and are not routinely available in health facilities in low-or middle-income countries where HDP are common.

Pregnant women with a history of hypertensive disorders in a previous pregnancy, those having chronic kidney disease, autoimmune disease, diabetes mellitus, or chronic hypertension, as well as nulliparous women, advanced age, or obese women, and those having multiple pregnancies, or family history of PE, were considered as risk factors of being advised to take aspirin for prevention of PE by the National Institute for Health and Care Excellence (NICE) 2019 and the American College of Obstetricians and Gynecologists’ Committee [15, 16]. To date, only aspirin has been recommended for PE prophylaxis in women with risk factors in the NICE guideline and the US Preventive Services Task Force recommendation [15, 17], not the other medications reported in previous systematic reviews [4–9]. The objectives of this analysis were to determine the relative effectiveness and provide a ranking of the available medications for preventing hypertensive disorders in high-risk pregnant women classified by the NICE 2019 using a network meta-analysis.

## Methods

### Eligibility criteria

We included all randomized controlled trials or cluster-randomized trials comparing the most commonly used medications by any route or doses in high-risk women during pregnancy for preventing hypertensive disorders. Only one main publication/report of the studies was selected to be reviewed and analyzed. Eligibility criteria were the studies that included pregnant women, at any gestational age, and at high risk of developing hypertensive disorders based on one of these following risk factors: nulliparity, family history of PE, history of pregnancy-induced hypertension in a previous pregnancy, obstetric risks (advanced maternal age, obesity, or multiple pregnancies), and underlying medical diseases (polycystic ovarian syndrome, autoimmune diseases, chronic renal diseases, diabetes, or chronic hypertension) in which medications were commenced only during pregnancy.

The studies were eligible if they used any of these groups of medications (antiplatelet agents, anticoagulants, antioxidants, nitric oxide, or calcium supplements) for preventing HDP and compared them against each other, placebo, or no treatment/conventional management. We considered medications routinely prescribed during pregnancy, such as ferrous, folic, or multivitamin supplementation, as conventional standard treatments. The medications prescribed before conception and continued during pregnancy were excluded. Two-arm or multi-arm trials that compared drug(s) in different dosages or regimens in the same medication group were included, if the comparison of medication groups could be made after the drug(s) in the same medication group were combined. Both primary outcomes (PE, gestational hypertension (GHT), and chronic hypertension with superimposed preeclampsia (SPE)) and secondary outcomes (placental abruption, postpartum hemorrhage, neonatal intraventricular hemorrhage, and neonate with small gestational age or growth restriction) were included in the protocol registered in PROSPERO [18]. However, in this network meta-analysis, the primary outcomes on relative effectiveness were focused, and the secondary outcomes on safety will be reported in other separate review with network meta-analysis.

### Information sources and search strategy

We received the search results from the database of the Cochrane Pregnancy and Childbirth’s Specialised Register of Controlled Trials, on 31st July 2020, using the topic area of “hypertension, prevention,” as the assigned search. This is a database, containing the results of over 30 years of searching for trials related to pregnancy and childbirth as a whole.

The full search methods, including individual strategies for each database search, can be found within the Trials Register section of the group’s webpage (https://pregnancy.cochrane.org/pregnancy-and-childbirth-groups-trials-register). The register is stored in the Cochrane Register of Studies. Each review receives its own specific search results, and no language was restricted.

### Selection and data collection process

Two review authors (TL, YY) screened the titles and abstracts of all search results independently, considering the criteria for included studies using the RAYYAN web-based application. Any discrepancies were solved by discussion. Two pairs of review authors (TL-YY, TL-CK) assessed the full texts independently to decide which of all the potential studies would be included using an electronic checklist form. We resolved any disagreements through discussion or in consultation with an independent reviewer (EO, RM), if required. A study flow diagram of the Preferred Reporting Items for Systematic reviews and Meta-Analyses (PRISMA) was used to present the number of records identified, excluded, or included.

We reviewed all included reports; however, if more than one reports come from the same study, we chose one main primary report as the main cited reference which the data were extracted for this review to avoid the data duplication. At least two of the review authors (TL, YY, CK, RM, EO) independently assessed the risk of bias for each study, using the criteria in the *Cochrane Handbook for Systematic Reviews of Interventions* [19]. TL and CK independently extracted the data.

### Study risk-of-bias assessment and certainty assessment

The criteria for assessing risk of bias included random sequence generation, allocation concealment, blinding, incomplete outcome data, selective reporting, other bias, and overall risk of bias. TL and CK assessed the quality of the evidence, using the Grading of Recommendations Assessment, Development and Evaluation (GRADE) methods [20]. Any disagreements were resolved by discussion, and the information was entered into Review Manager 5 software, for risk of bias [21]. The summary of findings of each outcome was presented using the template of GRADE network meta-analysis–summary of findings (NMA-SoF) tables for multiple treatment comparisons compared with placebo [22].

### Effect measures

We evaluated the assumption of transitivity epidemiologically, by comparing the clinical and methodological characteristics of sets of studies grouped by treatment comparisons. The drugs in the same two-arm or multiple-arm trials that are in the same groups of medication of interest in this review were grouped to be the same treatment node, regardless of regimens or doses. When the trials had more than one drugs in different treatment nodes in one arm, we defined them as the combinations group of medications. A network plot was drawn with the nodes representing interventions, the size of the nodes representing sample sizes, and the thickness of the lines connecting between nodes indicating the number of direct comparisons between pairs of interventions. A separate network plot was presented for primary outcomes on PE, GHT, and SPE.

We evaluated the inconsistency of the evidence on the network using the global inconsistency test [23] and the Dias’s side-splitting approach [24]. The heterogeneity of pairwise studies in the meta-analysis was assessed using the *I*^2^ statistic. If substantial heterogeneity, *I*^2^ > 50%, was identified, subgroup analysis considering different high-risk characteristics was explored [23, 25]. The comparative risk ratios (RR) and 95% confidence interval (CI) were estimated for pooled direct evidence, using a random-effects model and network meta-analysis using multivariate random-effects models. We estimated the surface under the cumulative ranking curve (SUCRA) to provide a hierarchy of the medications in numerical presentations. The SUCRA values ranged from 0 to 100%, with values close to 0 indicating a higher likelihood that a medication is in one of the bottom ranks, while values close to 100% indicate a higher likelihood that a medication is in one of the top ranks [26]. We also assessed the publication bias using a comparison-adjusted funnel plot and the Egger’s test, if at least 10 studies with the same comparisons and outcome were found because the power of the test is usually low to differentiate the chance of real asymmetry in fewer than 10 studies [27, 28].

The data were analyzed using STATA 15, with the “network” commands (The StataCorp, Texas, USA). Multivariate random-effects models were used to analyze both direct and indirect pairwise comparisons and network meta-analysis. The visualizations of RR and 95% CI of effect size of pairwise and network meta-analyses as well as ranking treatments among medications were operated in R software (R version 3.6.1, R Core Team 2019, Vienna, Austria), with “tidyverse,” “ggplot2,” “gridExtra,” and “RColorBrewer” packages. We reported this systematic review in accordance with the recommendations in PRISMA 2020 [29].

## Results

### Study selection

**Fig. 1:**
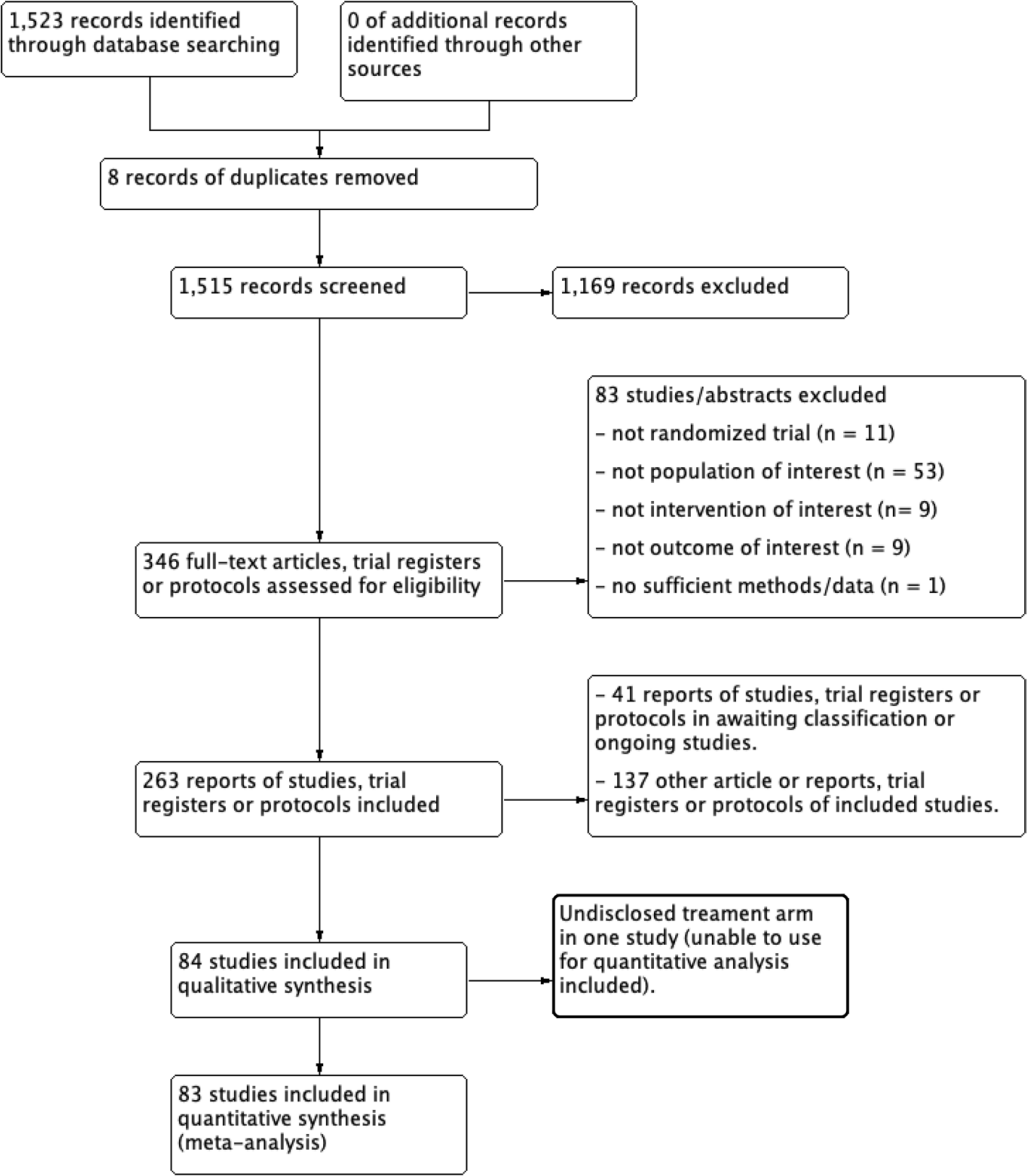
PRISMA study flow diagram

From 84 studies, there were 6998 women with outcomes of interest among 93,971 included women (7.4%). One included study, conducted in Columbia, comparing 100 mg aspirin with a placebo did not disclose the drug groups (drug 1, *n* = 54) and drug 2, *n* = 43) in the results of the study; hence, we could not use the data from this study for the analysis [30]. The results of search and selection process are presented in the PRISMA flow diagram as shown in Fig. 1.

### Study characteristics and risk of bias

**Fig. 2:**
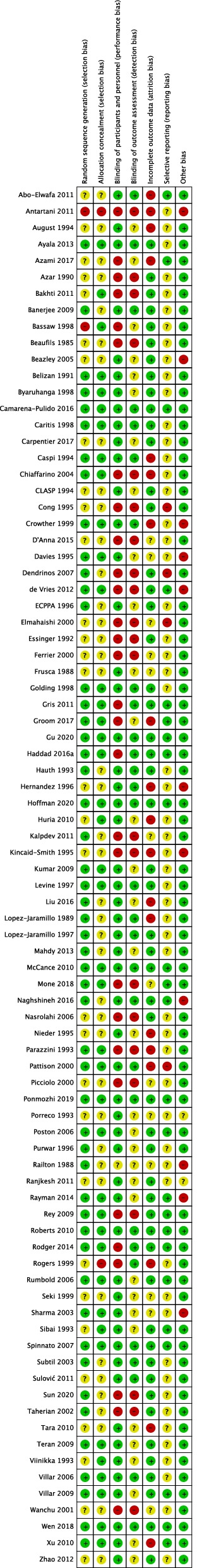
Risk-of-bias graph presented as percentages across all included studies

Among 83 studies with the outcomes of interest in this network meta-analysis, 77 studies reported PE [31–107], 39 studies reported gestational hypertension [31, 34, 36, 37, 39, 40, 42, 50, 51, 57, 58, 60, 61, 63, 66, 68, 69, 71, 72, 77, 81–83, 86, 88, 90, 91, 94, 96, 97, 102, 103, 106–112], and four studies reported SPE [56, 58, 112, 113]. The incidences of PE, GHT, and SPE in control groups using a placebo or no treatment were 7.8% (3559/45,449), 14.9% (4463/30,002), and 1.4% (45/3174), respectively. Risks-of-bias domain is summarized across all studies and presented in Fig. 2; 38 studies were judged to have a low risk of bias [34, 38, 42–45, 50, 51, 53, 58–64, 67, 68, 70, 72–74, 78, 80, 82, 86–89, 91, 94, 95, 101–103, 105, 106, 108]. There was no evidence of global inconsistency in the network analysis for all primary outcomes on PE, GHT, and SPE.

### Results of synthesis and certainty of evidence

**Fig. 3:**
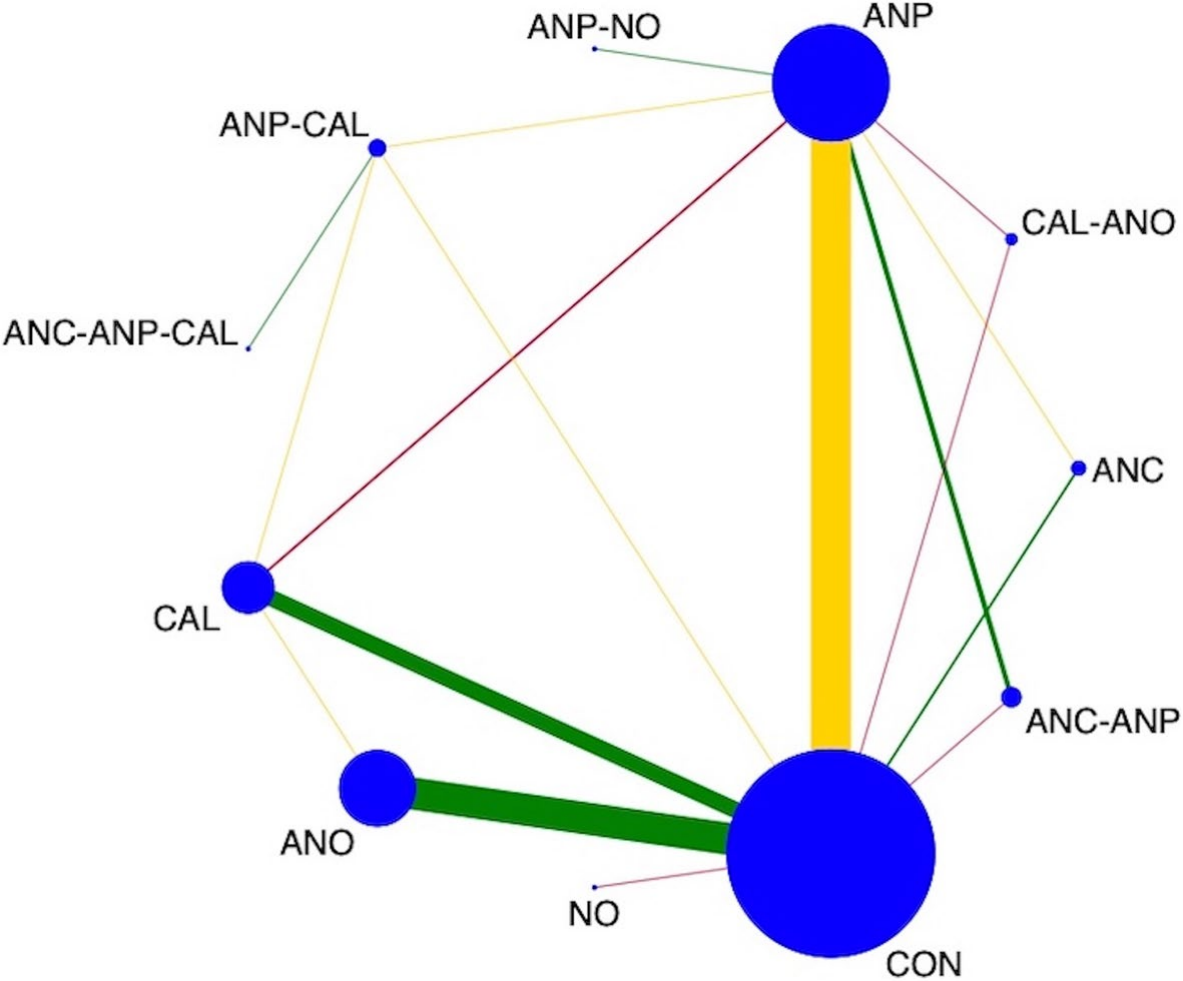
Network plot of medications for preventing preeclampsia. CON, control; ANC, anticoagulants; ANO, antioxidants; ANP, antiplatelet agents; CAL, calcium; N, nitric oxide; CAL-ANO, calcium plus antioxidants; ANC-ANP, anticoagulants plus antiplatelet agents; ANP-NO, antiplatelet agents plus nitric oxide; ANP-CAL, antiplatelet agents plus calcium; ANC-ANP-CAL, anticoagulants plus antiplatelet plus calcium

**Fig. 4:**
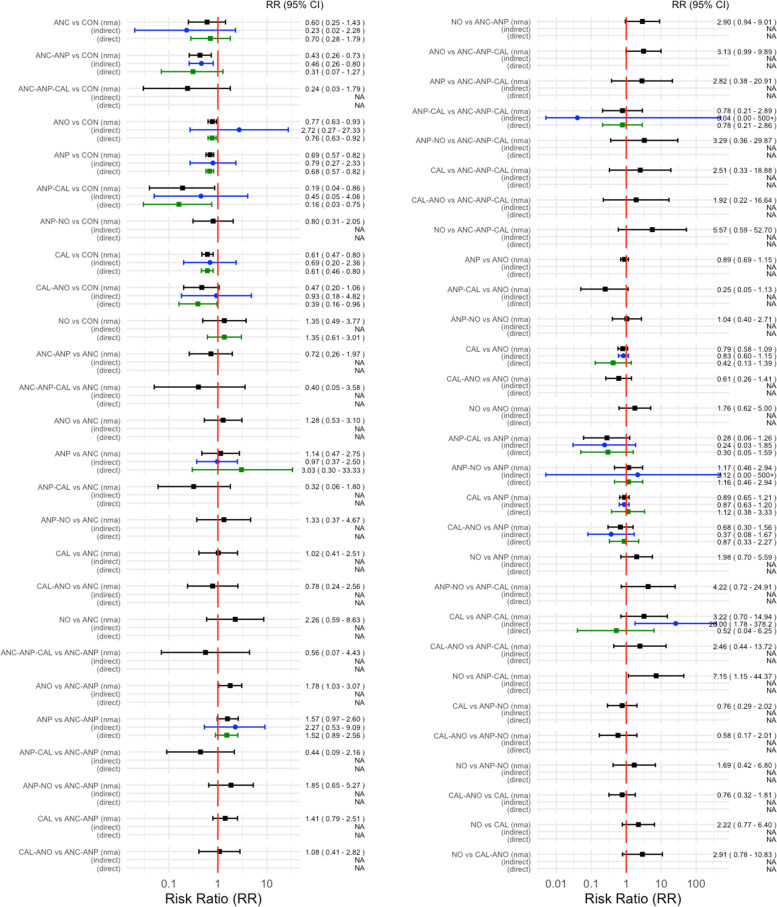
Direct, indirect, and network meta-analysis estimates of medications for preventing preeclampsia. CON, control; ANC, anticoagulants; ANO, antioxidants; ANP, antiplatelet agents; CA, calcium; NO, nitric oxide; CAL-ANO, calcium plus antioxidants; ANC-ANP, anticoagulants plus antiplatelet agents; ANP-NO, antiplatelet agents plus nitric oxide; ANP-CAL, antiplatelet agents plus calcium; ANC-ANP-CAL, anticoagulants plus antiplatelet plus calcium

The network diagram of 77 studies for preventing PE in all high-risk women is presented in Fig. 3. Antiplatelet agents were the most frequently investigated medications, in 38 of 77 studies (49.4%), followed by antioxidants in 25 studies (32.4%), calcium in 14 studies (18.2%), and various combinations in nine studies (11.7%). Pooled effect sizes, from direct estimates as well as network meta-analysis, are presented in Fig. 4. Calcium, antiplatelet agents, and combinations of antiplatelet agents with calcium probably had a moderately preventive effect for PE when compared with a placebo or no treatment as the evidence from network analysis accounted for antiplatelet agents with calcium (*RR* 0.19, 95% *CI* 0.04 to 0.86; 1 study; 334 participants; low-quality evidence); calcium (RR 0.61, 95% *CI* 0.47 to 0.80; 13 studies; 26,021 participants; moderate-quality evidence); antiplatelet agents (*RR* 0.69, 95% *CI* 0.57 to 0.82; 31 studies; 41,953 participants; moderate-quality evidence); and antioxidants (*RR* 0.77, 95% *CI* 0.63 to 0.93; 25 studies; 24,768 participants; moderate-quality evidence).

**Fig. 5:**
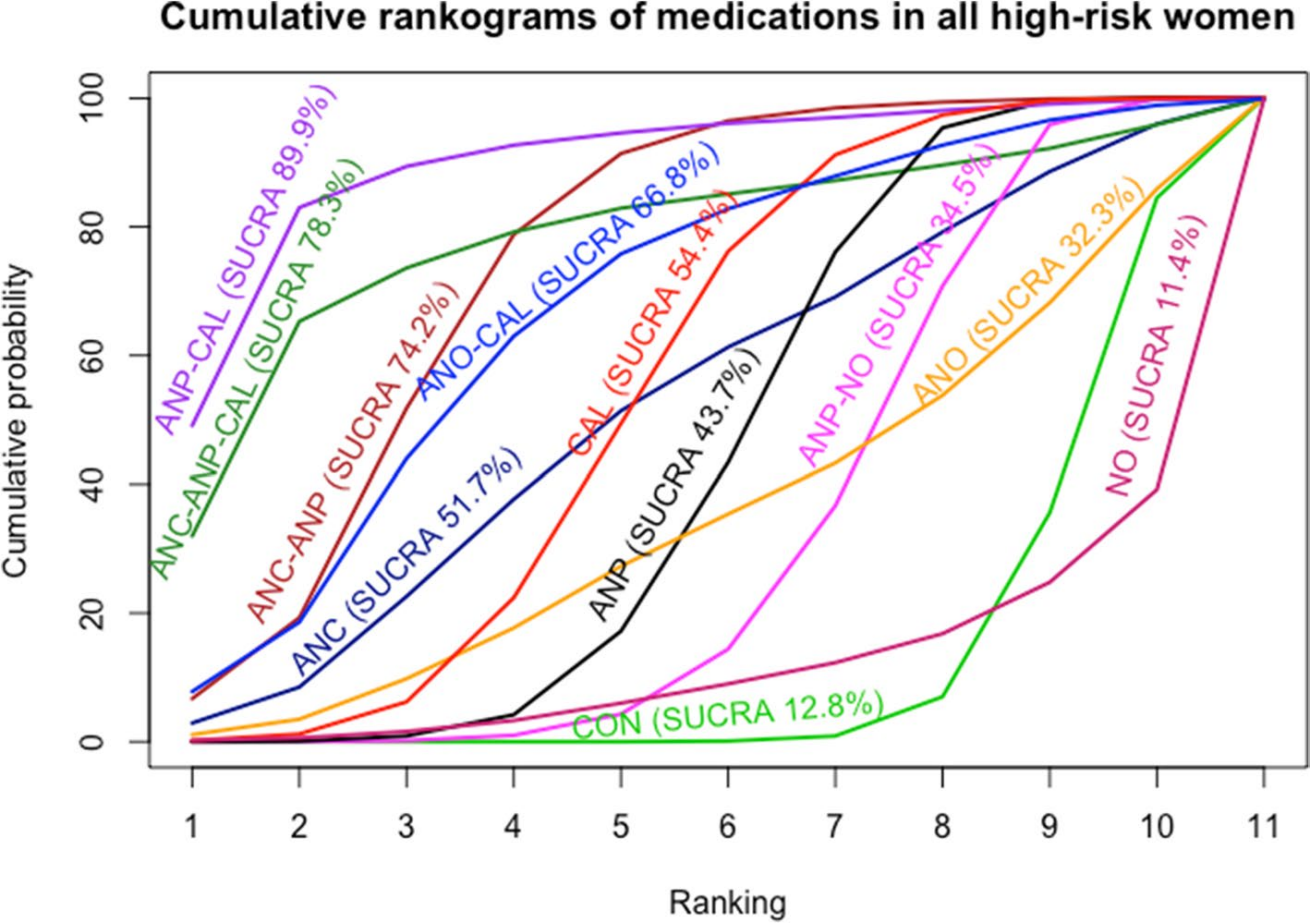
Cumulative rankograms of medications for preventing preeclampsia. CON, control; ANC, anticoagulants; ANO, antioxidants; ANP, antiplatelet agents; CAL, calcium; NO, nitric oxide; CAL-ANO, calcium plus antioxidants; ANC-ANP, anticoagulants plus antiplatelet agents; ANP-NO, antiplatelet agents plus nitric oxide; ANP-CAL, antiplatelet agents plus calcium; ANC-ANP-CAL, anticoagulants plus antiplatelet plus calcium

Antiplatelet agents with calcium in all high-risk women reported in one study showed highest SUCRA (89.9%) (Fig. 5). For the consistency of evidence on the network, the global inconsistency test was not significant (*P* = 0.459). The direct and indirect comparison estimates of each treatment pair by the Dias’s side splitting are presented in Fig. 4, and no significant treatment pairs were detected by the Dias’s inconsistency tests. The summary of findings for medication to prevent PE in all high-risk women is presented in Table 1. Certainty of evidence of the medications compared with a placebo or no treatment to prevent PE ranged from very low to moderate. Due to substantial heterogeneity (*I*^2^ 59.0%), subgroup analyses based on the high-risk subgroup population were performed, and the findings are shown in the summary of findings for subgroups on prevention of PE (Additional file 1: Appendices 1–3).

**Table 1:** Summary of findings for medications to prevent preeclampsia

Patient or population: Pregnant women at any gestational age at high risk of developing hypertensive disorders in pregnancy Settings: Hospital setting Intervention: Antiplatelet agents, anticoagulants, antioxidants, calcium, nitric oxide, and their combinations Comparator: Placebo or no treatment Outcome: Preeclampsia								
Total studies: 77 RCTs Total participants: 93,234	Direct estimates RR (95% ***CI***)	Certainty of evidence	Indirect estimates RR (95% ***CI***)	Certainty of evidence	Network estimates RR (95% ***CI***) [95% ***PrI***]	Certainty of evidence	SUCRA	Comments
Antiplatelets + calcium (1 RCT; 334 participants)	0.16 (0.03 to 0.75)	⊕⊕⊝⊝ Low^a,b^	0.45 (0.05 to 4.06)	⊕⊕⊕⊝ Moderate^b^	0.19 (0.04 to 0.86) [0.04 to 1.01]	⊕⊕⊝⊝ Low^a,b^	89.9%	There was no evidence of inconsistency for global inconsistency test (*P* = 0.459) and Dias’s inconsistency tests of the node splitting
Anticoagulants + antiplatelets + calcium (2 RCTs; 156 participants)	Not estimable		0.24 (0.03 to 1.79)	⊕⊝⊝⊝ Very low^c,d^	0.24 (0.03 to 1.79) [0.03 to 2.07]	⊕⊝⊝⊝ Very low^c,d^	78.3%	
Anticoagulants + antiplatelets (1 RCT; 20 participants)	0.31 (0.07 to 1.27)	⊕⊝⊝⊝ Very low^d,e^	0.46 (0.26 to 0.80)	⊕⊕⊝⊝ Low^d^	0.43 (0.26 to 0.73) [0.19 to 1.01]	⊕⊕⊝⊝ Low^d^	74.2%	
Calcium + antioxidants (1 RCT; 660 participants)	0.39 (0.16 to 0.96)	⊕⊕⊝⊝ Low^e^	0.93 (0.18 to 4.82)	⊕⊕⊕⊝ Moderate^b^	0.47 (0.20 to 1.06) [0.16 to 1.35]	⊕⊕⊕⊝ Moderate^b^	66.8%	
Calcium (13 RCTs; 26,021 participants)	0.61 (0.46 to 0.80)	⊕⊕⊕⊝ Moderate^f^	0.69 (0.20 to 2.36)	⊕⊕⊕⊝ Moderate^b^	0.61 (0.47 to 0.80) [0.30 to 1.24]	⊕⊕⊕⊝ Moderate^f^	54.4%	
Anticoagulants (2 RCTs; 399 participants)	0.70 (0.28 to 1.79)	⊕⊕⊕⊝ Moderate^b^	0.23 (0.02 to 2.28)	⊕⊕⊕⊝ Moderate^b^	0.60 (0.25 to 1.43) [0.20 to 1.80]	⊕⊕⊕⊝ Moderate^b^	51.7%	
Antiplatelets (31 RCTs; 41,953 participants)	0.68 (0.57 to 0.82)	⊕⊕⊝⊝ Low^a,f^	0.79 (0.27 to 2.33)	⊕⊕⊕⊝ Moderate^b^	0.69 (0.57 to 0.82) [0.35 to 1.35]	⊕⊕⊕⊝ moderate^f^	43.7%	
Antiplatelets + nitric oxide (No direct comparison)	Not estimable		Not estimable		0.80 (0.31 to 2.05) [0.25 to 2.55]	⊕⊕⊝⊝ low^d^	34.5%	
Antioxidants (25 RCTs; 24,768 participants)	0.76 (0.63 to 0.92)	⊕⊕⊕⊝ Moderate^f^	2.72 (0.27 to 27.33)	⊕⊕⊕⊝ Moderate^b^	0.77 (0.63 to 0.93) [0.39 to 1.52]	⊕⊕⊕⊝ Moderate^f^	32.3%	
Nitric oxide (1 RCT; 68 participants)	1.35 (0.61 to 3.01)	⊕⊝⊝⊝ Very low^d,e^	Not estimable		1.35 (0.49 to 3.77) [0.39 to 4.65]	⊕⊝⊝⊝ Very low^d,e^	11.4%	

GRADE Working Group grades of evidence

High quality: Further research is very unlikely to change our confidence in the estimate of effect

Moderate quality: Further research is likely to have an important impact on our confidence in the estimate of effect and may change the estimate

Low quality: Further research is very likely to have an important impact on our confidence in the estimate of effect and is likely to change the estimate

Very low quality: We are very uncertain about the estimate

The corresponding risk (and its 95% confidence interval) is based on the assumed risk in the comparison group and the relative effect of the intervention (and its 95% CI)

*CI* Confidence interval, *PrI* Prediction interval, *RR* Relative risk

^a^We downgraded (1) level for serious limitations in study design due to most of the studies being at unclear risk of bias

^b^We downgraded (1) level for serious imprecision due to wide confidence interval

^c^We downgraded (1) level for serious intransitivity due to without closed loop of intervention

^d^We downgraded (2) level for very serious imprecision due to wide confidence interval and small number of events and sample size

^e^We downgraded (2) level for very serious limitations in study design due to most of the studies being at high risk of bias

^f^We downgraded (1) level for serious publication bias due to asymmetry funnel plot and *P*-value of Egger’s test < 0.05

**Fig. 6:**
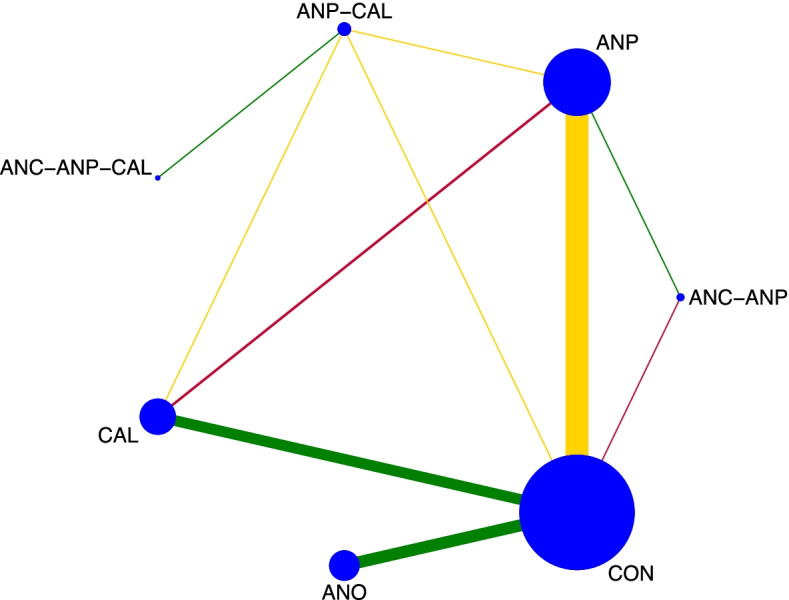
Network plot of medications for preventing gestational hypertension. CON, control; ANO, antioxidants; ANP, antiplatelet agents; CAL, calcium; ANC-ANP, anticoagulants plus antiplatelet agents; ANP-CAL, antiplatelet agents plus calcium; ANC-ANP-CA, anticoagulants plus antiplatelet plus calcium

**Fig. 7:**
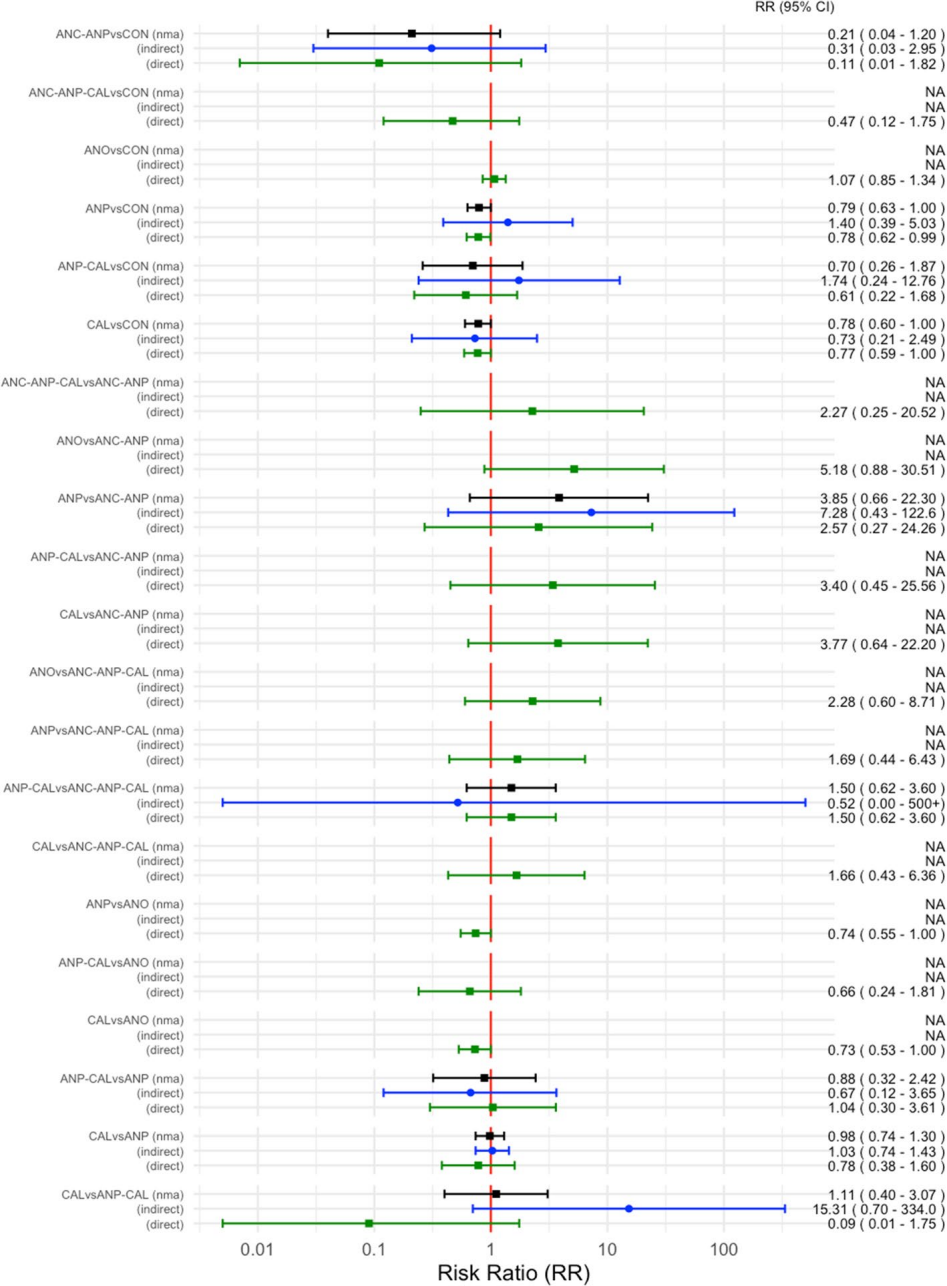
Direct, indirect, and network meta-analysis estimates of medications for preventing gestational hypertension. CON, control; ANO, antioxidants; ANP, antiplatelet agents; CAL, calcium; ANC-ANP, anticoagulants plus antiplatelet agents; ANP-CAL, antiplatelet agents plus calcium; ANC-ANP-CAL, anticoagulants plus antiplatelet plus calcium

**Fig. 8:**
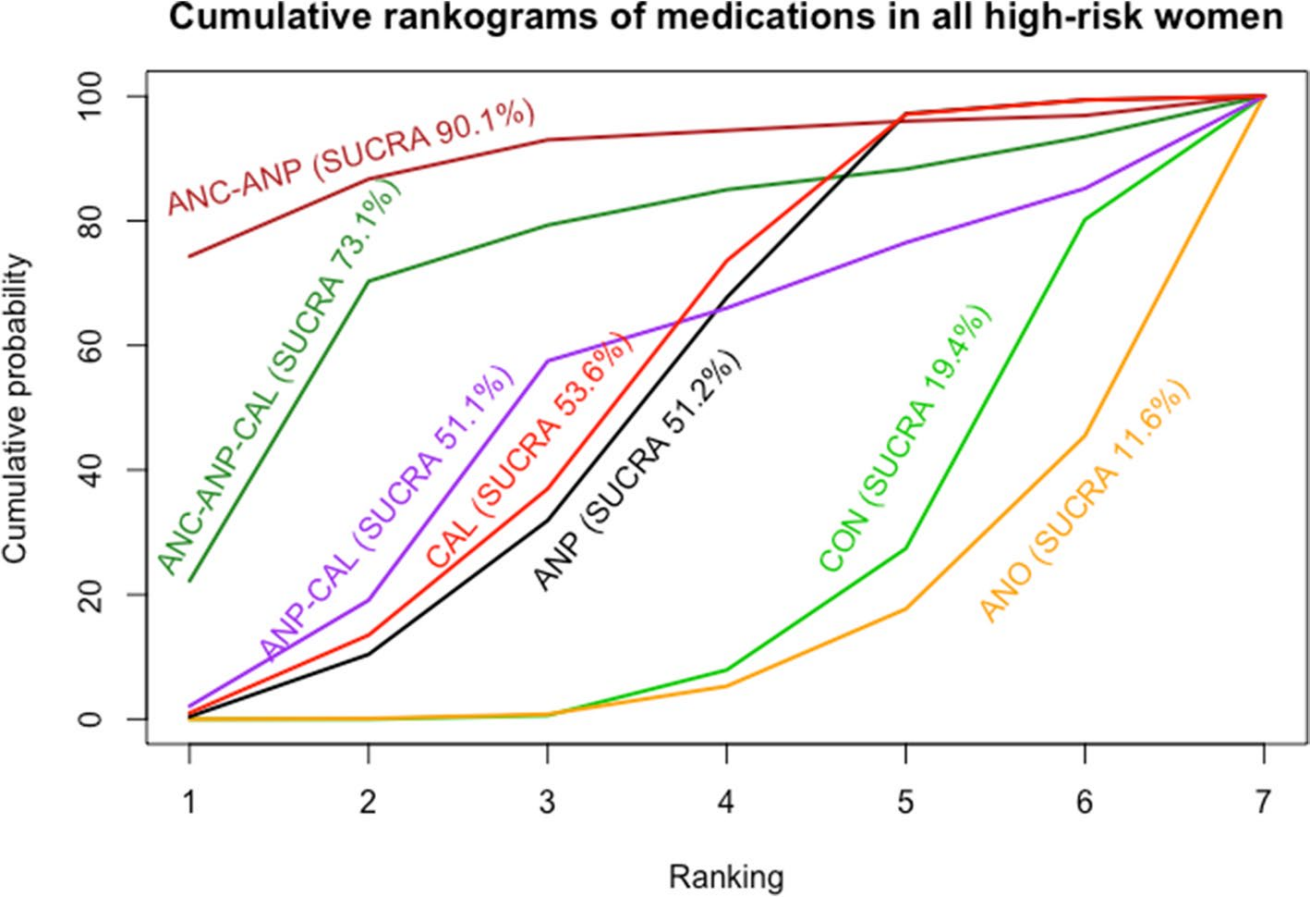
Cumulative rankograms of medications for preventing gestational hypertension. CON, control; ANO, antioxidants; ANP, antiplatelet agents; CAL, calcium; ANC-ANP, anticoagulants plus antiplatelet agents; ANP-CAL, antiplatelet agents plus calcium; ANC-ANP-CAL, anticoagulants plus antiplatelet plus calcium

The network diagram of 39 studies for preventing gestational hypertension is presented in Fig. 6. Antiplatelet agents were the most frequently investigated medications in 19 out of 39 studies (48.7%), followed by antioxidants in 10 studies (25.6%) and calcium in nine studies (23.1%). Pooled effect sizes from direct estimates as well as network meta-analysis (Fig. 7) suggested antiplatelet agents (*RR* 0.78, 95% *CI* 0.62 to 0.99 from direct estimates and *RR* 0.80, 95% *CI* 0.64 to 1.00 from network meta-analysis; 19 studies; 16,813 participants; moderate-quality evidence) or calcium (*RR* 0.77, 95% *CI* 0.59 to 1.00 from direct estimates and *RR* 0.78, 95% *CI* 0.61 to 1.00 from network meta-analysis; 9 studies; 24,534 participants; moderate-quality evidence) may prevent GHT. It is the uncertain effect of a combination of antiplatelet agents with anticoagulants in network meta-analysis estimate (*RR* 0.21, 95% *CI* 0.04 to 1.20; 1 study; 20 participants; very low-quality evidence) with highest SUCRA (90.1%) to prevent GHT in all high-risk women, as shown in Fig. 8.

For the consistency of evidence on the network, the global inconsistency test was not significant (*P* = 0.512). The direct and indirect comparison estimates of each treatment pair by the Dias’s side splitting are presented in Fig. 7, and no significant treatment pairs were detected by the Dias’s inconsistency tests. Summary of findings for medication to prevent gestational hypertension in all high-risk women is presented in Table 2. Certainty of evidence of the medications compared with a placebo or no treatment to prevent GHT ranged from very low to moderate. Due to substantial heterogeneity (*I*^2^ 63.2%), the subgroup analyses based on the high-risk subgroup population were performed, and the findings are shown in the table of summary of findings for subgroups on prevention of gestational hypertension (Additional file 1: Appendices 4–6).

**Table 2:** Summary of findings for medications to prevent gestational hypertension

Patient or population: Pregnant women at any gestational age at high risk of developing hypertensive disorders in pregnancy Settings: Hospital setting Intervention: Antiplatelet agents, anticoagulants, antioxidants, calcium, nitric oxide, and their combinations Comparator: Placebo or no treatment Outcome: Gestational hypertension								
Total studies: 39 RCTs Total participants: 60,953	Direct estimates RR (95% ***CI***)	Certainty of evidence	Indirect estimates RR (95% ***CI***)	Certainty of evidence	Network estimates RR (95% CI) [95% ***PrI***]	Certainty of evidence	SUCRA	Comments
Anticoagulants + antiplatelets (1 RCT; 20 participants)	0.11 (0.007 to 0.50)	⊕⊝⊝⊝ Very low^a,b^	0.31 (0.03 to 2.98)	⊕⊕⊝⊝ Low^c^	0.21 (0.04 to 1.20) [0.03 to 1.39]	⊕⊝⊝⊝ Very low^a,c^	90.1%	There was no evidence of inconsistency for global inconsistency test (*P* = 0.512) and Dias’s inconsistency tests of the node splitting
Anticoagulants + antiplatelets + calcium (1 RCT; 149 participants)	Not estimable		Not estimable		0.47 (0.13 to 1.74) [0.11 to 2.05]	⊕⊕⊕⊝ Low^c^	73.1%	
Calcium (9 RCTs; 24,534 participants)	0.77 (0.59 to 1.00)	⊕⊕⊕⊝ Moderate^b^	0.75 (0.22 to 2.53)	⊕⊕⊕⊝ Moderate^b^	0.78 (0.61 to 1.00) [0.43 to 1.39]	⊕⊕⊕⊝ Moderate^b^	53.6%	
Antiplatelets (19 RCTs; 16,813 participants)	0.78 (0.62 to 0.99)	⊕⊕⊕⊝ Moderate^d^	1.41 (0.40 to 5.00)	⊕⊕⊕⊝Moderate^b^	0.80 (0.64 to 1.00) [0.44 to 1.41]	⊕⊕⊕⊝ Moderate^b^	51.2%	
Antiplatelets + calcium (1 RCT; 334 participants)	0.61 (0.22 to 1.68)	⊕⊕⊝⊝ Low^b,d^	1.78 (0.24 to 12.92)	⊕⊕⊕⊝ Moderate^b^	0.70 (0.26 to 1.87) [0.22 to 2.22]	⊕⊕⊝⊝ Low^b,d^	51.1%	
Antioxidants (10 RCTs; 53,057 participants)	1.06 (0.85 to 1.34)	⊕⊕⊕⊝ Moderate^b^	Not estimable		1.07 (0.85 to 1.34) [0.60 to 1.90]	⊕⊕⊕⊝ Moderate^b^	11.6%	

GRADE Working Group grades of evidence

High quality: Further research is very unlikely to change our confidence in the estimate of effect

Moderate quality: Further research is likely to have an important impact on our confidence in the estimate of effect and may change the estimate

Low quality: Further research is very likely to have an important impact on our confidence in the estimate of effect and is likely to change the estimate

Very low quality: We are very uncertain about the estimate

The corresponding risk (and its 95% confidence interval) is based on the assumed risk in the comparison group and the relative effect of the intervention (and its 95% CI)

*CI* Confidence interval, *PrI* Prediction interval, *RR* Relative risk

^a^We downgraded (2) level for very serious limitations in study design due to most of the studies being at high risk of bias

^b^We downgraded (1) level for serious imprecision due to wide confidence interval

^c^We downgraded (2) level for very serious imprecision due to wide confidence interval and small number of events and sample size

^d^We downgraded (1) level for serious limitations in study design due to most of the studies being at unclear risk of bias

**Fig. 9:**
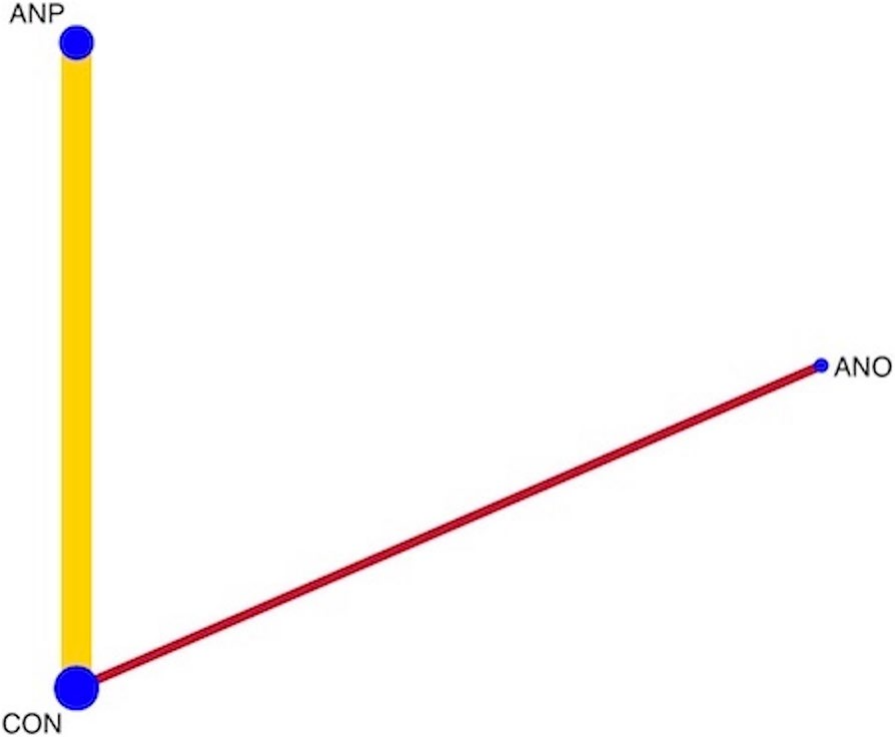
Network plot of medications for preventing superimposed preeclampsia. CON, control; ANO, antioxidants; ANP, antiplatelet agents

The network diagram of four studies for preventing SPE in all high-risk women is presented in Fig. 9. Pooled effect sizes from the network meta-analysis of four studies suggested the uncertainty of the evidence on antiplatelet agents when compared with a placebo or no treatment in network meta-analysis (*RR* 0.72, 95% *CI* 0.46 to 1.14; 3 study; 6298 participants; low-quality evidence). The summary of findings for medications in the prevention of SPE is presented in Table 3. Certainty of evidence of the medications compared with a placebo or no treatment to prevent SPE was very low or low. The inconsistency test using side-splitting approach was significant for SPE.

**Table 3:** Summary of findings for medications to prevent superimposed preeclampsia

Patient or population: Pregnant women at any gestational age at high risk of developing hypertensive disorders in pregnancy Settings: Hospital setting Intervention: Antiplatelet agents, anticoagulants, antioxidants, calcium, nitric oxide, and their combinations Comparator: Placebo or no treatment Outcome: Superimposed preeclampsia								
Total studies: 4 RCTs Total participants: 6,342	Direct estimates RR (95% ***CI***)	Certainty of evidence	Indirect estimates RR (95% ***CI***)	Certainty of evidence	Network estimates RR (95% ***CI***) [95% ***PrI***]	Certainty of evidence	SUCRA	Comments^a^
Antiplatelets (3 RCTs; 6,298 participants)	0.72 (0.46 to 1.14)	⊕⊕⊝⊝ Low^b,c^	Not estimable		0.72 (0.46 to 1.14) [0.04 to 14.21]	⊕⊕⊝⊝ Low^b,c^	69.0%	There was no evidence of inconsistency for global inconsistency test (*P* = 0.165)
Antioxidants (1 RCT; 44 participants)	0.67 (0.12 to 3.61)	⊕⊝⊝⊝ Very low^d,e^	Not estimable		0.67 (0.12 to 3.61) [< 0.001 to 37900]	⊕⊝⊝⊝ Very low^d,e^	60.6%	

GRADE Working Group grades of evidence

High quality: Further research is very unlikely to change our confidence in the estimate of effect

Moderate quality: Further research is likely to have an important impact on our confidence in the estimate of effect and may change the estimate.

Low quality: Further research is very likely to have an important impact on our confidence in the estimate of effect and is likely to change the estimate.

Very low quality: We are very uncertain about the estimate

The corresponding risk (and its 95% confidence interval) is based on the assumed risk in the comparison group and the relative effect of the intervention (and its 95% CI)

*CI* Confidence interval, *PrI* Prediction interval, *RR* Relative risk

^a^Dias’s inconsistency tests of the node splitting not estimable

^b^We downgraded (1) level for serious limitations in study design due to most of the studies being at unclear risk of bias

^c^We downgraded (1) level for serious imprecision due to wide confidence interval

^d^We downgraded (2) level for very serious limitations in study design due to most of the studies being at high risk of bias

^e^We downgraded (2) level for very serious imprecision due to wide confidence interval and small number of events and sample size

### Reporting biases

The summary on the tests of heterogeneity, effect of intervention, and tests of publication bias for direct comparisons in a network meta-analysis are presented for all primary outcomes (Additional file 1: Appendix 7). Publication biases, using comparison-adjusted funnel plot for preventing PE and GHT, were found with a *P*-value of Egger’s test < 0.001 (Additional files 2 and 3).

## Discussion

This network meta-analysis found that antiplatelet agents, calcium, antioxidants, and their combinations were more effective medications for preventing hypertensive disorders in pregnancy than a placebo or no treatment in different women’s contexts. It was uncertain that one medication was superior to the others. The qualities of evidence were rated to be moderate, due to the limitation of risk of bias, publication bias, or imprecision. There is the potential for medication combinations, such as antiplatelet agents with calcium, anticoagulants with antiplatelet agents, or calcium with antioxidants, to be slightly better, but evidence was limited with only few current studies and large confidence intervals. More studies investigating these combination treatments are needed.

The effectiveness of antiplatelet agents and calcium on prevention of PE was similar to the findings of two previous systematic reviews and a meta-analysis [4, 7]. Doses of antiplatelet agents used in the included studies in this network meta-analysis ranged from 50 to 150 mg daily aspirin or 300 mg dipyridamole. For calcium, the daily doses ranged from 1000 to 2000 mg elemental calcium. Our findings support the WHO guidelines of 2011, which strongly recommends 1.5–2.0 g elemental calcium/day in areas where dietary calcium intake is low, or 75 mg of aspirin for the prevention of PE in women at high risk of developing the condition with moderate quality of evidence [114], and the NICE recommendation for the use of 75–150 mg aspirin [15]. The majority of antioxidants used were a combination of daily 1000 mg vitamin C plus 200–400 mg vitamin E. Our network meta-analysis found a preponderance of evidence that antioxidants could reduce PE and gestational hypertension, although this finding was opposite to the finding of a previous systematic review [115]. The combinations of antiplatelet agents with calcium or antioxidants with calcium, and antiplatelet agents with anticoagulants, had high cumulative probabilities for being the highest rank for preventing PE and/or GHT with low- to moderate-quality evidence, even though the studies were small. More research on combining antiplatelet agents with calcium may be needed.

The findings of our network meta-analysis were consistent with the results of two previous network meta-analyses, which found that calcium supplementation could reduce the risk of PE; however, these systematic reviews did not rate the quality of evidence using GRADE [10, 11]. In addition, the first review did not clearly describe risk characteristics of women in the results [10], and the latter defined nulliparous women as low-risk women [11]. The probability of being the most effective treatment for calcium in our review was higher than that in the study of Sanchez-Ramos (2017) [10]. The effectiveness of antiplatelet agents in our review supports the suggestion of using aspirin prophylaxis for PE from a previous systematic review and meta-analysis [116]. However, the qualities of evidence for the outcomes in our review were rated as ranging from very low to moderate. These were then downgraded, due to the risk of bias, imprecision, and publication bias regarding a GRADE approach.

There were limitations of this network meta-analysis. First, a wide range of high-risk pregnant women were included, resulting in the heterogeneous findings of included studies. This may be explained by different responses to the medications in various risk characteristics. Second, we focused on the studies conducted in hospital settings using high-risk factors suggested by NICE 2019, not Doppler, laboratory tests, or serum markers for screening risk of hypertensive disorders in pregnancy. Third, the subgroup analysis on intervention (different drugs in the same group of medication in the intervention arm, different doses of the same drug, or gestational age at the time the medication was given) and gestational age at the time the outcome occurred was not performed in this network meta-analysis. Fourth, this review presented parts of the results on relative effectiveness of medications, and safety outcomes will be reported in a separate review. Both aspects of effectiveness and safety are essential to consider the benefits outweigh the risks of medications to pregnant women. Lastly, PE with preterm birth was not included as the outcome in this network analysis.

## Conclusions

Antiplatelet agents, calcium, antioxidants, and their combinations were more effective medications than a placebo or no treatment for preventing hypertensive disorders in different risks of pregnant women’s context. It was uncertain that one medication was superior to the others. The combinations of antiplatelet agents with calcium or anticoagulants were in one of the top ranks to prevent PE; however, the evidence was limited due to imprecision and heterogeneity leading to different clinical decisions in a future study. Calcium was in one of the top ranks to prevent GHT in nulliparous or primigravida women. Further network meta-analyses considering different drugs in the same groups of medications, different doses of the same drug, gestational age at the time the medications are given, and gestational age at the time the outcome occurred are required, so as to identify the most effective regimen of drugs for preventing hypertensive disorders in pregnancy.

## Supplementary Information

**Additional file 1: Appendix 1.** Summary of findings for medications to prevent pre-eclampsia in subgroup: the studies including high-risk women with underlying diseases. **Appendix 2.** Summary of findings for medications to prevent pre-eclampsia in subgroup: the studies including high-risk women with no underlying diseases or mixed nulliparous women and women with no underlying diseases. **Appendix 3.** Summary of findings for medications to prevent pre-eclampsia in subgroup: the studies including nulliparous or primigravida women. **Appendix 4.** Summary of findings for medications to prevent gestational hypertension in subgroup: studies including high-risk women with underlying diseases or mixed other high-risk women. **Appendix 5.** Summary of findings for medications to prevent gestational hypertension in subgroup: the studies including high-risk women with no underlying diseases or mixed nulliparous women and women with no underlying diseases. **Appendix 6.** Summary of findings for medications to prevent gestational hypertension in subgroup: the studies including nulliparous or primigravida women. **Appendix 7.** Findings on the tests of heterogeneity, effect of intervention and tests of publication bias for direct comparisons in a network meta-analysis.

**Additional file 2.** Publication biases using comparison-adjusted funnel plot for preventing preeclampsia. 01: anticoagulants; 02: anticoagulants plus antiplatelet agents; 03: anticoagulants plus antiplatelet plus calcium; 04: antioxidants; 05: antiplatelet agents; 06: antiplatelet agents plus calcium; 07: antiplatelet agents plus nitric oxide; 08: calcium; 09: calcium plus antioxidants; 10: control; 11: nitric oxide.

**Additional file 3.** Publication biases using comparison-adjusted funnel plot for preventing gestational hypertension. 01: anticoagulants plus antiplatelet agents; 02: anticoagulants plus antiplatelet plus calcium; 03: antioxidants; 04: antiplatelet agents; 05: antiplatelet agents plus calcium; 06: calcium; 07: control.

## Data Availability

The datasets used and/or analyzed during the current study are available from the corresponding author on reasonable request.

## Abbreviations

CI: Confidence interval
GHT: Gestational hypertension
GRADE: Grading of Recommendations Assessment, Development and Evaluation
HDP: Hypertensive disorders in pregnancy
NICE: National Institute for Health and Care Excellence
NMA-SoF: Network meta-analysis–summary of findings
PE: Preeclampsia
PRISMA: Preferred Reporting Items for Systematic reviews and Meta-Analyses
RR: Risk ratios
SUCRA: Surface under the cumulative ranking curve
SPE: Superimposed preeclampsia
