# Supplementary material for: Medications for preventing hypertensive disorders in high-risk pregnant women: a systematic review and network meta-analysis
Source: Syst Rev. 2022 Jul 1;11:135. doi: 10.1186/s13643-022-01978-5 (PMC9250249; doi:10.1186/s13643-022-01978-5)
Supplement: Supplementary file 1 — Additional file 1: Appendix 1. Summary of findings for medications to prevent pre-eclampsia in subgroup: the studies including high-risk women with underlying diseases. Appendix 2. Summary of findings for medications to prevent pre-eclampsia in subgroup: the studies including high-risk women with no underlying diseases or mixed nulliparous women and women with no underlying diseases. Appendix 3. Summary of findings for medications to prevent pre-eclampsia in subgroup: the studies including nulliparous or primigravida women. Appendix 4. Summary of findings for medications to prevent gestational hypertension in subgroup: studies including high-risk women with underlying diseases or mixed other high-risk women. Appendix 5. Summary of findings for medications to prevent gestational hypertension in subgroup: the studies including high-risk women with no underlying diseases or mixed nulliparous women and women with no underlying diseases. Appendix 6. Summary of findings for medications to prevent gestational hypertension in subgroup: the studies including nulliparous or primigravida women. Appendix 7. Findings on the tests of heterogeneity, effect of intervention and tests of publication bias for direct comparisons in a network meta-analysis. [file 13643_2022_1978_MOESM1_ESM.pdf]

Appendix 1 Summary of findings for medications to prevent pre-eclampsia in subgroup: the studies including high-risk women with underlying diseases

Patient or population: high-risk pregnant women with underlying diseases at any gestational age
Settings: hospital setting
Intervention: antiplatelet agents, anticoagulants, antioxidants, calcium, nitric oxide, and their combinations
Comparator: placebo or no treatment
Outcome: pre-eclampsia

**Total studies:** 6 RCTs<br>**Total participants:** 1,197	**Direct estimates RR (95% CI)**	**Certainty of evidence**	**Indirect estimates RR (95% CI)**	**Certainty of evidence**	**Network estimates RR (95% CI)***	**Certainty of evidence**	**SUCRA**	**Comments †**
Anticoagulants + Antiplatelets (1 RCT; 20 participants)	0.31 (0.09 to 1.09)	⊕⊖⊖⊖ Very low[1,2]	Not estimable		0.31 (0.06 to 1.47)	⊕⊖⊖⊖ Very low[1,2]	87.7%	There was no evidence of inconsistency for global approach (p = 0.144).
Antioxidants (1 RCT; 749 participants)	0.81 (0.59 to 1.12)	⊕⊕⊖⊖ Low[2]	Not estimable		0.81 (0.31 to 2.16)	⊕⊕⊖⊖ Low[2]	49.0%	
Antiplatelets (3 RCTs; 139 participants)	0.66 (0.26 to 1.69)	⊕⊖⊖⊖ Very low[2,3]	4.70 (0.40 to 55.22)	⊕⊕⊖⊖ Low[2]	0.89 (0.30 to 2.67)	⊕⊕⊖⊖ Low[2]	43.2%	
Anticoagulants (1 RCT; 289 participants)	1.57 (0.53 to 4.67)	⊕⊕⊖⊖ Low[2]	0.22 (0.02 to 2.42)	⊕⊕⊖⊖ Low[2]	1.01 (0.26 to 4.00)	⊕⊕⊖⊖ Low[2]	36.7%	

The corresponding risk (and its 95% confidence interval) is based on the assumed risk in the comparison group and the relative effect of the intervention (and its 95% CI). CI: Confidence interval; RR: Relative Risk
*Prediction interval: not estimable
†Significant inconsistency tests of the node splitting

GRADE Working Group grades of evidence
High quality: Further research is very unlikely to change our confidence in the estimate of effect.
Moderate quality: Further research is likely to have an important impact on our confidence in the estimate of effect and may change the estimate.
Low quality: Further research is very likely to have an important impact on our confidence in the estimate of effect and is likely to change the estimate.
Very low quality: We are very uncertain about the estimate.

Footnotes
[1]We downgraded (2) level for very serious limitation in study design due to most of the studies being at high risk of bias.
[2]We downgraded (2) level for very serious imprecision due to wide confidence interval and small number of events and sample size.
[3]We downgraded (1) level for serious limitation in study design due to most of the studies being at unclear risk of bias.

Appendix 2 Summary of findings for medications to prevent pre-eclampsia in subgroup: the studies including high-risk women with no underlying diseases or mixed nulliparous women and women with no underlying diseases

Patient or population: high-risk pregnant women with no underlying diseases or mixed nulliparous women and women with no underlying diseases
Settings: hospital setting
Intervention: antiplatelet agents, anticoagulants, antioxidants, calcium, nitric oxide, and their combinations
Comparator: placebo or no treatment
Outcome: pre-eclampsia

**Total studies:** 10 RCTs<br>**Total participants:** 1,850	**Direct estimates RR (95% CI)**	**Certainty of evidence**	**Indirect estimates RR (95% CI)**	**Certainty of evidence**	**Network estimates RR (95% CI) [95% PrI]**	**Certainty of evidence**	**SUCRA**	**Comments**
Calcium<br>(1 RCT; 331 participants)	0.08<br>(0.01 to 0.63)	⊕⊕⊖⊖<br>Low[1,2]	0.08<br>(0.005 to 1.43)	⊕⊕⊕⊖<br>Moderate[2]	0.08<br>(0.01 to 0.60)<br>[<0.001 to 28970]	⊕⊕⊖⊖<br>Low[1,2]	88.1%	There was no evidence of inconsistency for global approach (p = 0.998).
Antiplatelets + Calcium<br>(1 RCT; 334 participants)	0.16<br>(0.04 to 0.70)	⊕⊕⊖⊖<br>Low[1,2]	0.16<br>(0.01 to 1.92)	⊕⊕⊕⊖<br>Moderate[2]	0.16<br>(0.04 to 0.65)<br>[<0.001 to 1387]	⊕⊕⊖⊖<br>Low[1,2]	77.4%	
Anticoagulants + Antiplatelets + Calcium<br>(no direct comparison)	Not estimable		Not estimable		0.21<br>(0.03 to 1.25)<br>[<0.001 to 24978]	⊕⊕⊖⊖<br>Low[3]	64.5%	
Anticoagulants<br>(1 RCT; 110 participants)	0.27<br>(0.08 to 0.93)	⊕⊕⊖⊖<br>Low[3]	Not estimable		0.27<br>(0.08 to 0.92)<br>[<0.001 to 745.56]	⊕⊕⊖⊖<br>Low[3]	59.4%	
Anticoagulants + Antiplatelets (no direct comparison)	Not estimable		Not estimable		0.36<br>(0.19 to 0.67)<br>[0.006 to 19.98]	⊕⊕⊖⊖<br>Low3	52.1%	
Antioxidants<br>(1 RCT; 40 participants)	0.53<br>(0.31 to 0.90)	⊕⊖⊖⊖<br>Low[1,2]	Not estimable		0.53<br>(0.32 to 0.89)<br>[0.02 to 15.24]	⊕⊖⊖⊖<br>Low[1,2]	30.1%	
Antiplatelets†<br>(5 RCTs; 918 participants)	0.54<br>(0.35 to 0.84)	⊕⊕⊕⊖<br>Moderate[1]	1.40<br>(<0.001 to >1000)	⊕⊕⊕⊖<br>Moderate[2]	0.54<br>(0.32 to 0.84)<br>[0.03 to 9.22]	⊕⊕⊕⊖<br>Moderate[1]	27.2%	

The corresponding risk (and its 95% confidence interval) is based on the assumed risk in the comparison group and the relative effect of the intervention (and its 95% CI). CI: Confidence interval; PrI: Prediction interval; RR: Relative Risk
†Significant inconsistency tests of the node splitting

GRADE Working Group grades of evidence
High quality: Further research is very unlikely to change our confidence in the estimate of effect.
Moderate quality: Further research is likely to have an important impact on our confidence in the estimate of effect and may change the estimate.
Low quality: Further research is very likely to have an important impact on our confidence in the estimate of effect and is likely to change the estimate.
Very low quality: We are very uncertain about the estimate.

Footnotes
[1]We downgraded (1) level for serious limitations in study design due to most of the studies being at unclear risk of bias.
[2]We downgraded (1) level for serious imprecision due to wide confidence interval.
[3]We downgraded (2) level for very serious imprecision due to wide confidence interval and small number of events and sample size.

Appendix 3 Summary of findings for medications to prevent pre-eclampsia in subgroup: the studies including nulliparous or primigravida women

Patient or population: nulliparous women or primigravida at any gestational age
Settings: hospital setting
Intervention: antiplatelet agents, anticoagulants, antioxidants, calcium, nitric oxide, and their combinations
Comparator: placebo or no treatment
Outcome: pre-eclampsia

**Total studies:** 32 RCTs **Total participants:** 66,836	**Direct estimates RR (95% CI)**	**Certainty of evidence**	**Indirect estimates RR (95% CI)**	**Certainty of evidence**	**Network estimates RR (95% CI) [95% PrI]**	**Certainty of evidence**	**SUCRA**	**Comments**
Calcium + Antioxidants (1 RCT; 660 participants)	0.39 (0.15 to 1.05)	⊕⊖⊖⊖ Very low[1,2]	1.14 (0.17 to 7.53)	⊕⊕⊕⊖ Moderate[2]	0.48 (0.19 to 1.20) [0.14 to 1.70]	⊕⊕⊖⊖ Moderate[2]	79.7%	There was no evidence of inconsistency for global approach (p = 0.357) and Dias's inconsistency tests of the node splitting.
Calcium (11 RCTs; 25311 participants)	0.62 (0.45 to 0.85)	⊕⊕⊕⊖ Moderate[3]	4.75 (0.81 to 27.95)	⊕⊕⊕⊖ Moderate[2]	0.63 (0.46 to 0.86) [0.26 to 1.52]	⊕⊕⊕⊖ Moderate[3]	64.1%	
Antioxidants (11 RCTs; 42289 participants)	0.64 (0.45 to 0.93)	⊕⊕⊕⊖ Moderate[3]	Not estimable		0.64 (0.45 to 0.93) [0.26 to 1.59]	⊕⊕⊕⊖ Moderate[3]	60.9%	
Antiplatelets (11 RCTs; 27193 participants)	0.73 (0.53 to 1.02)	⊕⊕⊕⊖ Moderate[3]	0.40 (0.04 to 4.36)	⊕⊕⊕⊖ Moderate[2]	0.73 (0.53 to 1.01) [0.44 to 2.95]	⊕⊕⊕⊖ Moderate[3]	42.9%	

The corresponding risk (and its 95% confidence interval) is based on the assumed risk in the comparison group and the relative effect of the intervention (and its 95% CI). CI: Confidence interval; PrI: Prediction interval; RR: Relative Risk

GRADE Working Group grades of evidence
High quality: Further research is very unlikely to change our confidence in the estimate of effect.
Moderate quality: Further research is likely to have an important impact on our confidence in the estimate of effect and may change the estimate.
Low quality: Further research is very likely to have an important impact on our confidence in the estimate of effect and is likely to change the estimate.
Very low quality: We are very uncertain about the estimate.

Footnotes
[1]We downgraded (2) level for very serious limitations in study design due to most of the studies being at high risk of bias.
[2]We downgraded (1) level for serious imprecision due to wide confidence interval.
[3]We downgraded (1) level for serious publication bias due to asymmetry funnel plot and p value of Egger's test <0.05.

Appendix 4 Summary of findings for medications to prevent gestational hypertension in subgroup: studies including high-risk women with underlying diseases or mixed other high-risk women

**Patient or population:** high-risk pregnant women with underlying diseases at any gestational age<br>**Settings:** hospital setting<br>**Intervention:** antiplatelet agents, anticoagulants, antioxidants, calcium, nitric oxide, and their combinations<br>**Comparator:** placebo or no treatment<br>**Outcome:** gestational hypertension								
**Total studies:** 14 RCTs<br>**Total participants:** 9,621	**Direct estimates RR (95% CI)**	**Certainty of evidence**	**Indirect estimates RR (95% CI)**	**Certainty of evidence**	**Network estimates RR (95% CI) [95% PrI]**	**Certainty of evidence**	**SUCRA**	**Comments**
Anticoagulants + Antiplatelets (1 RCT; 20 participants)	0.11 (0.006 to 1.97)	⊕⊖⊖⊖ Very low[1,2]	0.23 (0.02 to 2.53)	⊕⊕⊖⊖ Low2	0.17 (0.03 to 1.08) [0.02 to 1.76]	⊕⊕⊖⊖ Low[2]	95.0%	There was no evidence of inconsistency for global approach (p = 0.692) and Dias's inconsistency tests of the node splitting.
Antiplatelets (7 RCTs; 7242 participants)	0.60 (0.38 to 0.95)	⊕⊕⊕⊖ Moderate[3]	0.28 (0.007 to 11.58)	⊕⊕⊕⊖ Moderate4	0.59 (0.38 to 0.94) [0.39 to 3.27]	⊕⊕⊕⊖ Moderate[3]	68.7%	
Antioxidants (5 RCTs; 8181 participants)	1.14 (0.96 to 1.34)	⊕⊕⊕⊖ Moderate[4]	Not estimable		1.13 (0.74 to 1.71) [0.20 to 1.76]	⊕⊕⊕⊖ Moderate[4]	11.0%	
The corresponding risk (and its 95% confidence interval) is based on the assumed risk in the comparison group and the relative effect of the intervention (and its 95% CI). CI: Confidence interval; PrI: Prediction interval; RR: Relative Risk								
GRADE Working Group grades of evidence<br>High quality: Further research is very unlikely to change our confidence in the estimate of effect.<br>Moderate quality: Further research is likely to have an important impact on our confidence in the estimate of effect and may change the estimate.<br>Low quality: Further research is very likely to have an important impact on our confidence in the estimate of effect and is likely to change the estimate.<br>Very low quality: We are very uncertain about the estimate.								
Footnotes<br>[1]We downgraded (2) level for very serious limitations in study design due to most of the studies being at high risk of bias.<br>[2]We downgraded (2) level for very serious imprecision due to wide confidence interval and small number of events and sample size.<br>[3]We downgraded (1) level for serious limitations in study design due to most of the studies being at unclear risk of bias.<br>[4]We downgraded (1) level for serious imprecision due to wide confidence interval.								

Appendix 5 Summary of findings for medications to prevent gestational hypertension in subgroup: the studies including high-risk women with no underlying diseases or mixed nulliparous women and women with no underlying diseases

Patient or population: high-risk pregnant women with no underlying diseases or mixed nulliparous/primigravida women at any gestational age
Settings: hospital setting
Intervention: antiplatelet agents, anticoagulants, antioxidants, calcium, nitric oxide, and their combinations
Comparator: placebo or no treatment
Outcome: gestational hypertension

**Total studies:** 7 RCTs<br>**Total participants:** 1,281	**Direct estimates RR (95% CI)**	**Certainty of evidence**	**Indirect estimates RR (95% CI)**	**Certainty of evidence**	**Network estimates RR (95% CI) ***	**Certainty of evidence**	**SUCRA**	**Comments†**
Calcium<br>(1 RCT; 334 participants)	0.06<br>(0.004 to 0.94)	⊕⊕⊕⊖<br>Moderate[1]	0.05<br>(0.002 to 1.41)	⊕⊕⊕⊖<br>Moderate[2]	0.06<br>(0.004 to 0.93)	⊕⊕⊕⊖<br>Moderate[1]	86.7%	There was no evidence of inconsistency for global approach (p = 0.880).
Antioxidants<br>(1 RCT; 220 participants)	0.08<br>(0.004 to 1.35)	⊕⊖⊖⊖<br>Very low[3,4]	Not estimable		0.08<br>(0.004 to 1.35)	⊕⊖⊖⊖<br>Very low[3,4]	81.6%	
Anticoagulants + Antiplatelets + Calcium<br>(no direct comparison)	Not estimable		Not estimable		0.41<br>(0.13 to 1.26)	⊕⊕⊖⊖<br>Low[4]	55.1%	
Antiplatelets<br>(5 RCTs; 1284 participants)	0.57<br>(0.41 to 0.78)	⊕⊕⊕⊖<br>Moderate[1]	2.17<br>(<0.001 to (>1000)	⊕⊕⊕⊖<br>Moderate[2]	0.57<br>(0.41 to 0.78)	⊕⊕⊕⊖<br>Moderate[1]	39.9%	
Antiplatelets + Calcium<br>(1 RCT; 334 participants)	0.61<br>(0.25 to 1.48)	⊕⊕⊖⊖<br>Low[1,2]	0.55<br>(0.08 to 3.98)	⊕⊕⊕⊖<br>Moderate[2]	0.61<br>(0.25 to 1.46)	⊕⊕⊕⊖<br>Moderate[2]	31.5%	

The corresponding risk (and its 95% confidence interval) is based on the assumed risk in the comparison group and the relative effect of the intervention (and its 95% CI). CI: Confidence interval; PrI: Prediction interval; RR: Relative Risk
*Prediction interval: not estimable
†Significant inconsistency tests of the node splitting

GRADE Working Group grades of evidence
High quality: Further research is very unlikely to change our confidence in the estimate of effect.
Moderate quality: Further research is likely to have an important impact on our confidence in the estimate of effect and may change the estimate.
Low quality: Further research is very likely to have an important impact on our confidence in the estimate of effect and is likely to change the estimate.
Very low quality: We are very uncertain about the estimate.

Footnotes
[1]We downgraded (1) level for serious limitations in study design due to most of the studies being at unclear risk of bias.
[2]We downgraded (1) level for serious imprecision due to wide confidence interval.
[3]We downgraded (2) level for very serious limitations in study design due to most of the studies being at high risk of bias.
[4]We downgraded (2) level for very serious imprecision due to wide confidence interval and small number of events and sample size.

Appendix 6 Summary of findings for medications to prevent gestational hypertension in subgroup: the studies including nulliparous or primigravida women

Patient or population: nulliparous women or primigravida at any gestational age
Settings: hospital setting
Intervention: antiplatelet agents, anticoagulants, antioxidants, calcium, nitric oxide, and their combinations
Comparator: placebo or no treatment
Outcome: gestational hypertension

**Total studies:** 18 RCTs<br>**Total participants:** 59,611	**Direct estimates RR (95% CI)**	**Certainty of evidence**	**Indirect estimates RR (95% CI)**	**Certainty of evidence**	**Network estimates RR (95% PrI)**	**Certainty of evidence**	**SUCRA**	**Comments**
Calcium†<br>(8 RCTs; 40,447 participants)	0.89<br>(0.84 to 0.95)	⊕⊕⊕⊕<br>High	1.12<br>(0.43 to 2.95)	⊕⊕⊕⊖<br>Moderate[1]	0.89<br>(0.84 to 0.95)<br>[0.83 to 0.96]	⊕⊕⊕⊕<br>High	99.9%	There was no evidence of inconsistency for global approach (p = 0.366).
Antiplatelets†<br>(7 RCTs; 49,739 participants)	1.05<br>(0.94 to 1.17)	⊕⊕⊕⊕<br>High	1.65<br>(0.63 to 1.33)	⊕⊕⊕⊖<br>Moderate[1]	1.05<br>(0.95 to 1.17)<br>[0.94 to 1.18]	⊕⊕⊕⊕<br>High	30.2%	
Antioxidants (4 RCTs; 34,616 participants)	1.08<br>(0.92 to 1.27)	⊕⊕⊕⊖<br>Moderate[1]	Not estimable		1.10<br>(1.03 to 1.17)<br>[1.03 to 1.17]	⊕⊕⊕⊖<br>Moderate[1]	8.7%	

The corresponding risk (and its 95% confidence interval) is based on the assumed risk in the comparison group and the relative effect of the intervention (and its 95% CI). CI: Confidence interval; PrI: Prediction interval; RR: Relative Risk
†Significant inconsistency tests of the node splitting

GRADE Working Group grades of evidence
High quality: Further research is very unlikely to change our confidence in the estimate of effect.
Moderate quality: Further research is likely to have an important impact on our confidence in the estimate of effect and may change the estimate.
Low quality: Further research is very likely to have an important impact on our confidence in the estimate of effect and is likely to change the estimate.
Very low quality: We are very uncertain about the estimate.

Footnotes
[1]We downgraded (1) level for serious imprecision due to wide confidence interval.

Appendix 7 Findings on the tests of heterogeneity, effect of intervention and tests of publication bias for direct comparisons in a network meta-analysis

Outcomes	Total studies	Test for heterogeneity		RR (95% CI) Random effect	P value of Egger's test
		I-squared	Tau		
**Pre-eclampsia**	**77**	**59%**	**0.069**	**0.73 (0.66-0.80)**	**<0.001**
Population subgroup by intervention					
UD	6	2.2%	0.006	0.78 (0.57-1.06)	
No UD	7	32.5%	0.098	0.59 (0.38-0.91)	
Primi	32	63.9%	0.074	0.71 (0.61-0.81)	
ANO vs CON		62.6%	0.144	0.65 (0.46-0.91)	
ANP vs CON		63.5%	0.086	0.77 (0.60-1.00)	
CAL vs CON		69.3%	0.078	0.66 (0.52-0.84)	
ANP vs CAL		NA	0.078	0.46 (0.12-1.69)	
ANO vs CAL_ANO		NA	0.078	1.15(0.56-2.39)	
CAL_ANO vs CON		NA	0.078	0.39 (0.21-0.74)	
Mixed UD	29	58.2%	0.055	0.78 (0.68-0.90)	
ANO vs CON		29.3%	0.014	0.94 (0.82-1.08)	
ANP vs CON		69.4%	0.09	0.66 (0.52-0.84)	
CAL vs CON		NA	0.09	0.36 (0.13-1.02)	
NO vs CON		NA	<0.001	1.35 (0.61-3.01)	
ANC_ANP vs ANP		0.0%	<0.001	0.81(0.42-1.55)	
ANO vs CAL		NA	0.09	2.50 (0.88-7.10)	
ANP vs ANP_NO		NA	<0.001	0.86 (0.44-1.66)	
Mixed No UD	3	61.9%	0.582	0.64 (0.31-1.32)	
ANP vs CON		0.0%	<0.001	0.53 (0.34-0.83)	
CAL vs CON		NA	<0.001	0.08 (0.01-0.60)	
ANP_CAL vs CON		NA	<0.001	0.16 (0.04-0.65)	
ANP vs ANP_CAL		NA	<0.001	3.38 (0.72-15.80)	
ANP vs CAL		NA	<0.001	6.52 (0.82-51.82)	

ANP_CAL vs CAL		NA	<0.001	1.93 (0.18-20.86)	
**Gestational hypertension**	**39**	**63.2%**	**0.034**	**0.90 (0.82-0.99)**	**<0.001**
Population subgroup by intervention					
UD	2	59.2%	1.481	0.52 (0.07-3.89)	
No UD	4	22.6%	0.234	0.37 (0.15-0.93)	
Primi	18	63.5%	0.018	0.95 (0.87-1.05)	
ANO vs CON		27.9%	0.008	1.08 (0.92-1.27)	
ANP vs CON		22.2%	0.008	1.04 (0.90-1.21)	
CAL vs CON		58.1%	0.014	0.85 (0.75-0.97)	
ANP vs CAL		NA	0.008	1.28 (0.74-2.21)	
Mixed UD	12	70.6%	0.128	0.83 (0.63-1.10)	
ANO vs CON		41.8%	0.019	1.17 (0.94-1.45)	
ANP vs CON		76.4%	0.377	0.56 (0.32-0.98)	
ANC_ANP vs ANP		NA	0.377	0.83 (0.63-1.10)	
Mixed No UD	3	40.0%	0.15	0.69 (0.44-1.10)	
**Superimposed pre-eclampsia**	**4**	**0.0%**	**<0.001**	**0.72 (0.46-1.12)**	**0.404**

UD: underlying diseases; No UD: without underlying diseases; Primi: primigradiva or nulliparous women; Mixed UD: mixed population from without to with underlying diseases; Mixed No UD: mixed population from primigravida or nulliparous women to without underlying diseases

CON: control; ANO: antioxidants; ANP: antiplatelet agents; CAL: calcium; NO: nitric oxide; CAL_ANO: calcium plus antioxidants; ANC_ANP: anticoagulants plus antiplatelet agents; ANP_NO: antiplatelet agents plus nitric oxide; ANP_CAL: antiplatelet agents plus calcium
