# Supplementary figures and images for: Medications for preventing hypertensive disorders in high-risk pregnant women: a systematic review and network meta-analysis
Source: Syst Rev. 2022 Jul 1;11:135. doi: 10.1186/s13643-022-01978-5 (PMC9250249; doi:10.1186/s13643-022-01978-5)

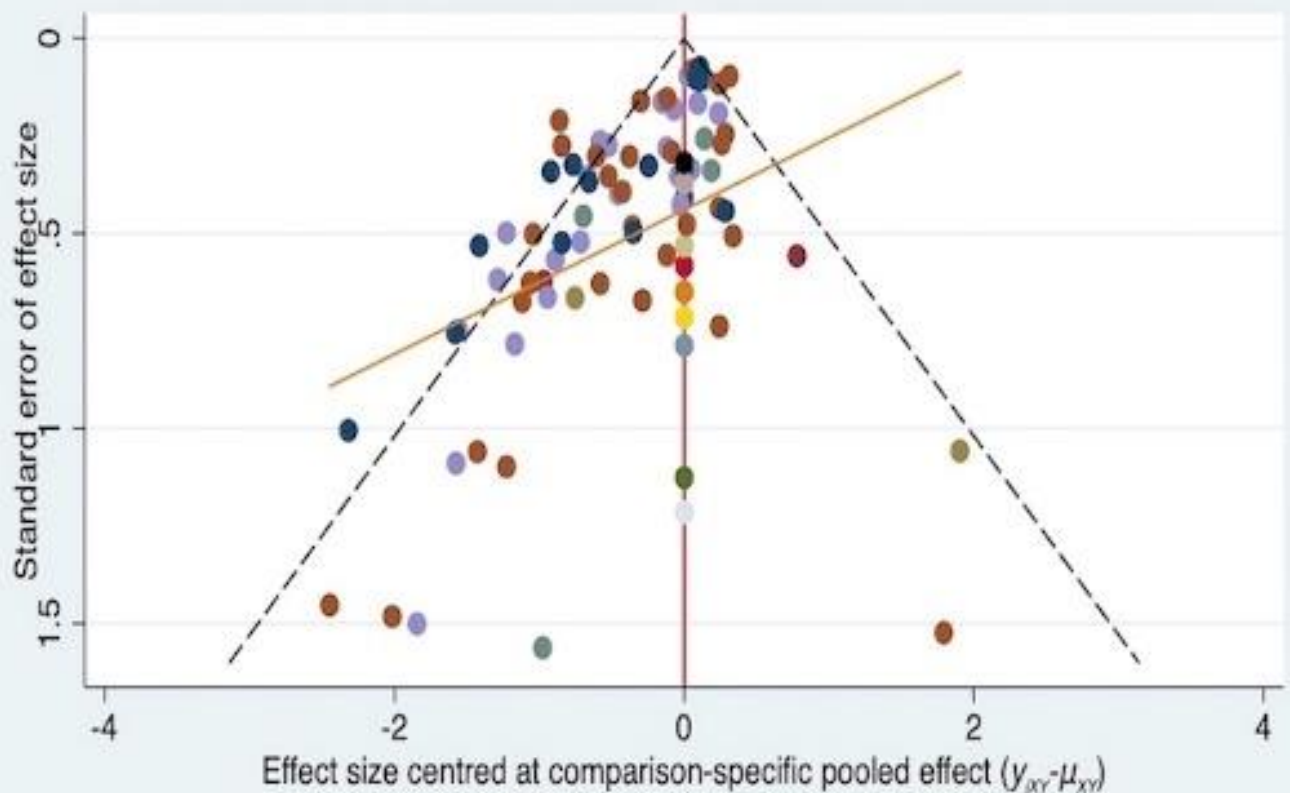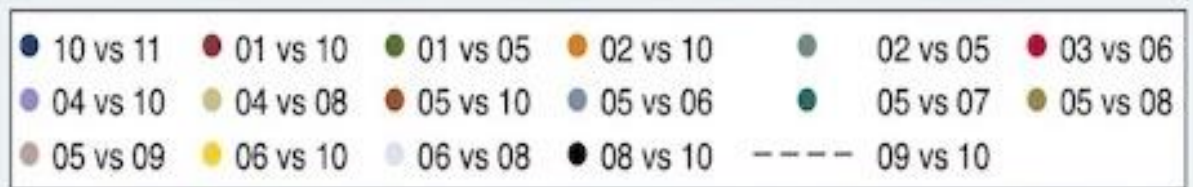

1-ANC; 2-ANC-ANP; 3-ANC-ANP-CAL; 4-ANO; 5-ANP; 6-ANP-CAL; 7-ANP-NO; 8-CAL; 9-CAL-ANO; 10-CON; 11-NO

Supplement: Supplementary file 2 — Additional file 2. Publication biases using comparison-adjusted funnel plot for preventing preeclampsia. 01: anticoagulants; 02: anticoagulants plus antiplatelet agents; 03: anticoagulants plus antiplatelet plus calcium; 04: antioxidants; 05: antiplatelet agents; 06: antiplatelet agents plus calcium; 07: antiplatelet agents plus nitric oxide; 08: calcium; 09: calcium plus antioxidants; 10: control; 11: nitric oxide. [file 13643_2022_1978_MOESM2_ESM.pdf]

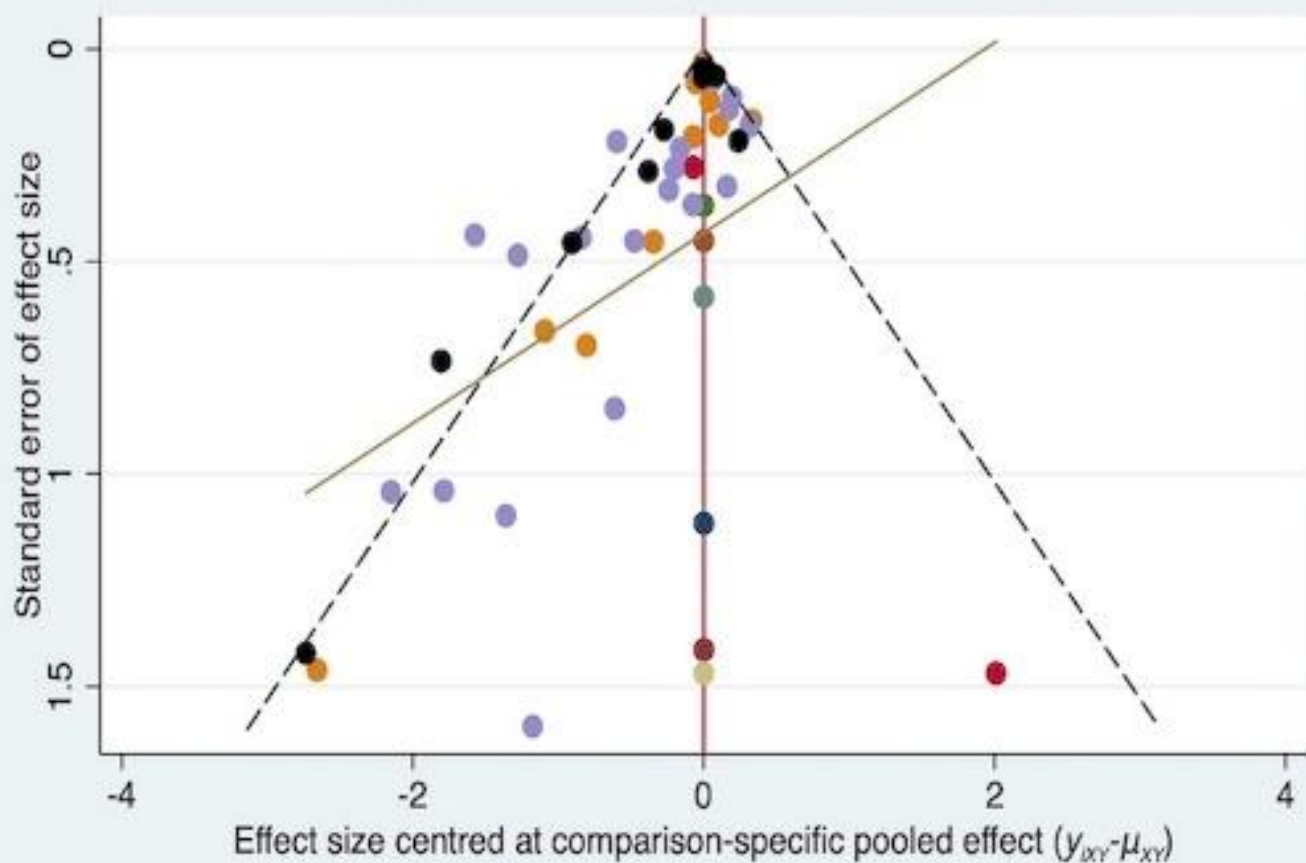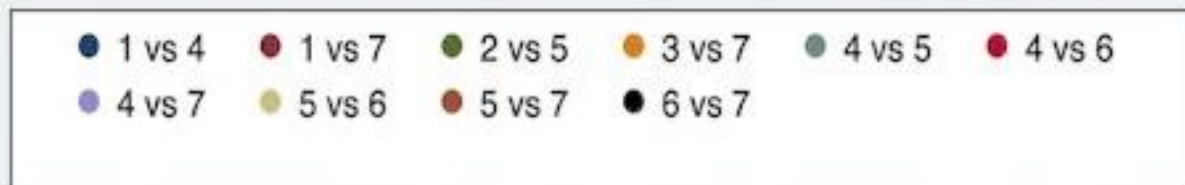

1-ANC-ANP; 2-ANC-ANP-CAL; 3-ANO; 4-ANP; 5-ANP-CAL; 6-CAL; 7-CON

Supplement: Supplementary file 3 — Additional file 3. Publication biases using comparison-adjusted funnel plot for preventing gestational hypertension. 01: anticoagulants plus antiplatelet agents; 02: anticoagulants plus antiplatelet plus calcium; 03: antioxidants; 04: antiplatelet agents; 05: antiplatelet agents plus calcium; 06: calcium; 07: control. [file 13643_2022_1978_MOESM3_ESM.pdf]
